# Life satisfaction in the context of the COVID-19 pandemic among middle school adolescents in France: findings from a repeated cross-sectional survey (EnCLASS, 2012–2021)

**DOI:** 10.3389/fped.2023.1204171

**Published:** 2023-08-08

**Authors:** Cynthia Hurel, Virginie Ehlinger, Michal Molcho, Jérémie F. Cohen, Bruno Falissard, Mariane Sentenac, Emmanuelle Godeau

**Affiliations:** ^1^ Department of Human and Social Sciences, EHESP School of Public Health, Rennes, France; ^2^ Rennes University, Centre Hospitalier Universitaire (CHU) de Rennes, Department of Epidemiology and Public Health, Rennes, France; ^3^ UMR 1295 CERPOP, Inserm, Université Toulouse III - Paul Sabatier, Team SPHERE, Toulouse, France; ^4^ Department of Children’s Studies, School of Education, University of Galway, Galway, Ireland; ^5^ Department of General Pediatrics and Pediatric Infectious Diseases, Hôpital Necker-Enfants malades, AP-HP, Université Paris Cité, Paris, France; ^6^ Université Paris Cité, Inserm, INRAE, Centre for Research in Epidemiology and StatisticS (CRESS), Obstetrical Perinatal and Pediatric Epidemiology Research Team, EPOPé, Paris, France; ^7^ Paris-Saclay University, UVSQ, CESP, Inserm U1018, Paris, France; ^8^ Public Health and Epidemiology Department, AP-HP, Hôpital du Kremlin Bicêtre, Le Kremlin Bicêtre, France

**Keywords:** life satisfaction, adolescents’, COVID-19, well - being, mental health, cross-sectional study, school survey

## Abstract

**Background and aims:**

Since the COVID-19 pandemic, several studies have reported a decrease in adolescents' well-being. We aim to describe life satisfaction over the last decade and examine the factors associated with its variations between 2020 and 2021 among French students in their last year of middle school (around 14–15 years old).

**Methods:**

Data were drawn from a repeated biennial cross-sectional national survey conducted in French schools over the last decade (EnCLASS study), using a self-administered questionnaire. After describing life satisfaction trends between 2012 and 2021 using the Cantril ladder, we examined individual changes in life satisfaction between 2020 and 2021 and their associations with housing and studying conditions during the COVID-19 lockdown, using multinomial logistic regression analysis (decrease, increase, no change as reference).

**Results:**

Among the 17,686 survey respondents, an overall slight decrease in the prevalence of adolescents reporting high life satisfaction (i.e., Cantril score ≥6) was observed since 2012 with the lowest proportion reported in 2021 (77.4%). Between 2020 and 2021, 16.3% of French adolescents experienced an improvement in life satisfaction, while 17.7% experienced the opposite. Decrease in life satisfaction between 2020 and 2021 was more likely experienced by adolescents living in reconstructed families [aOR 2.09 (95%CI, 1.58–2.77)], those who did not have their own room [aOR 1.58 (1.16–2.15)], nor access to the Internet to interact with their friends during the lockdown [aOR 1.47 (1.09–1.98)]. Interestingly, more girls than boys were represented in both those reporting increase and decrease in life satisfaction [aOR 1.82 (1.40–2.37) and 1.43 (1.14–1.79), respectively].

**Conclusions:**

This study shows that the way adolescents experienced the first 2020 lockdown in France was not uniform, and that one must consider sex as well as housing and studying conditions when interpreting adolescents' life satisfaction decrease during the COVID-19 pandemic.

## 1. Introduction

Many studies conducted since 2020 have suggested that the COVID-19 pandemic and its mitigation measures have had numerous psychosocial consequences, including on adolescents' well-being. More specifically, several studies found a decrease in adolescents' quality of life and life satisfaction, and an increase in the incidence of mental health disorders such as anxiety, depressive syndrome, psychosomatic complaints, and suicidal ideation (1–5). Authors identified protective and risk factors of well-being, associated with sociodemographic characteristics, social and family environment, physical environment, and lifestyle. Those findings on adolescents' well-being have been confirmed in France by the Confeado study, which aimed to understand how children and adolescents aged 9–16 years experienced the first lockdown in France (between March and May 2020), and how it may have affected their well-being (6). With 3,898 children and adolescents included, the Confeado study found that better living conditions during lockdown were associated with higher resilience scores. However, few studies have been able to contextualize the impact of the pandemic (and its mitigation measures) on adolescents' well-being and measure it over time (7).

Life satisfaction is a term related to subjective well-being and can be used to summarize how people perceive their life (8). It can be measured by the Cantril ladder, a one-item validated scale that is easy to understand and quick to answer, including by adolescents (8, 9). Life satisfaction among adolescents or young adults seems strongly correlated to life experience and mental health, especially the positive aspects of mental health (8, 10).

Based on data collected from adolescents periodically, the study EnCLASS is a unique opportunity to analyze data over the past decade to identify time trends in life satisfaction before and during the COVID-19 pandemic. Taking those trends into consideration, we further assessed associations between individual changes in life satisfaction among students in their last year of middle school (around 14–15 years) between 2020 and 2021 and housing and studying conditions during the COVID-19 lockdown experimented in March 2020 (measured retrospectively).

## 2. Materials and methods

### 2.1. Study design and sample

Our study used the data collected for the French national survey in middle- and high-school among adolescents on health and substances (EnCLASS). EnCLASS is a repeated biennial cross-sectional survey conducted in schools in France by self-questionnaire. It is a collaboration between two international surveys: Health Behaviour in School-aged Children (HBSC) (11, 12) and European School Survey Project on Alcohol and Other Drugs (ESPAD) (13). Through this partnership, researchers provide every two years since 2012 data from adolescents enrolled in the last year of middle school (corresponding to grade 9 of Junior High School in the United States). Surveys took place during spring 2012, 2014, 2016, 2018, and 2021.

To achieve a nationally representative sample of students enrolled in the last year of middle school in metropolitan France, a two-stage cluster sampling method was used for each survey, with schools as a primary sampling unit and classes as a secondary sampling unit. In order to guarantee equal probability of sampling at the student level, schools were selected with a probability proportional to their size, within the database of the Ministry of Education. Two classes were randomly selected in each middle school, and then invited to participate in the survey.

### 2.2. Data collection

At each survey wave, students completed confidential, anonymous, self-completion questionnaires, during dedicated classroom sessions.

#### 2.2.1. Life satisfaction

Since 2012, life satisfaction has been measured with the validated Cantril ladder (score 0–10; 10 meaning the best possible life) (8, 9). The full text of the question is as follows: “Here is a picture of a ladder. The top of the ladder ‘10’ is the best possible life for you and the bottom ‘0’ is the worst possible life for you. In general, where on the ladder do you feel you stand at the moment?”. In 2021, we also asked students retrospectively about their life satisfaction at the time of the first French COVID-19 national lockdown (March 15–May 11, 2020, see Supplementary Figure S1), with an adapted Cantril ladder: “Here is a picture of a ladder. The top of the ladder ‘10’ is the best possible life for you and the bottom ‘0’ is the worst possible life for you. Where on the ladder do you feel you stood *during the lockdown*?”. As in previous studies, the score was dichotomized, and respondents with a score of 6 or more were classified as reporting high life satisfaction (8, 14–16).

Further, students who participated in the 2021 survey were categorized into three life satisfaction groups according to their individual changes between 2020 and 2021: improved life satisfaction, no change in life satisfaction, and decreased life satisfaction. A 3-point threshold (greater than or equal to 3) was chosen to categorize the individual changes between 2020 and 2021, corresponding to one standard deviation of the distribution of the difference calculated for each student between the 2020 and 2021 scores.

#### 2.2.2. Sociodemographic characteristics

The following sociodemographic characteristics were collected at each survey wave: sex, educational delay, family structure, and parental employment. Students with chronic conditions were identified through a single-item developed for the HBSC survey and based on a non-categorical approach (17): “Do you have a long-term illness, disability, or medical condition (such as, diabetes, arthritis, allergy or cerebral palsy) that was diagnosed by a doctor?” (yes/no) (14). Family affluence level was measured using the HBSC family affluence scale (FAS) which included four items in 2012, and was revised in 2014 into a six-item scale to integrate changes in living conditions (18, 19). Due to the COVID-19 pandemic, the scale used in 2021 excluded the item asking students how many times the family went abroad for a holiday/vacation in the previous year. Family affluence scores were divided into tertiles defined within each survey wave, in order to allow comparisons between surveys: low- and high-affluence groups were defined as the lowest and the highest tertile, respectively, within each survey wave.

#### 2.2.3. Housing and studying conditions during lockdown

Housing and studying conditions during the lockdown of spring 2020 were retrospectively reported by students in the 2021 survey with yes-no questions asking them whether they: (1) had their own room, (2) had access to a screen to contact classmates or friends online and (3) had access to an outdoor space such as a balcony, a yard, a patio or a garden.

Students' perceptions of study conditions were retrospectively reported through the following item: “During this first lockdown, you were able to study in good conditions” with a 5-point Likert scale (Totally agree/Agree/Neither agree nor disagree/Disagree/Totally disagree). Then, responses were grouped into three categories: Good study conditions/Neither good nor bad study conditions/Bad study conditions.

### 2.3. Statistical analysis

In order to contextualize individual changes in life satisfaction between 2020 and 2021, we first examined life satisfaction trends between 2012 and 2021. Main sample characteristics were described for each survey year. To compare life satisfaction between survey waves, Poisson regression models with robust variance were computed with the survey year as a categorical variable (20, 21). The models were controlled for educational delay because this factor was both associated with life satisfaction and survey year, as practices in France regarding class repetition changed between 2012 and 2021. Results were presented as prevalence ratios with 95% confidence intervals (CI); they were derived from the models to compare the probability of high life satisfaction in each survey year to that in 2021. The possible modification effect due to interaction with vulnerability factors (chronic conditions, sex, FAS level) was examined each vulnerability factor independently.

Then, socioeconomic characteristics and living conditions during lockdown were described. We examined the associations between those factors and categories of changes in life satisfaction between 2020 (retrospective collection) and 2021. We used Chi-squared test with Rao & Scott's second-order correction and multinomial logistic regression models with change in life satisfaction as the response variable (decreased life satisfaction between 2020 and 2021 vs. no change; improved life satisfaction between 2020 and 2021 vs. no change). Adjusted models on sex and educational delay were fitted in order to obtain odds ratios (OR) of the associations with CI.

To correct for nonresponse at the school, class and student levels, calibration weights were used to ensure the representativeness of last year of middle school students, using data from the French Ministry of Education (calibration variables in 2014: class, sex and age; 2018: class, sex, sector (public/private), type of school's municipality (rural/urban); 2021: class, sex, sector). Statistical analyses were performed using Stata version 14.1 and R version 4.2.0 and took into account the calibration weights and the intra-class correlation due to the cluster design of each survey.

### 2.4. Ethics

Prior to participation, for each survey wave, adolescents and their parents were informed of the rationale of EnCLASS surveys and of their right to object to their participation in the survey. Each wave has been approved by the French national council for statistical information and has been declared to the French national information science and liberties commission.

## 3. Results

### 3.1. Trends in life satisfaction between 2012 and 2021

**Figure 1:**
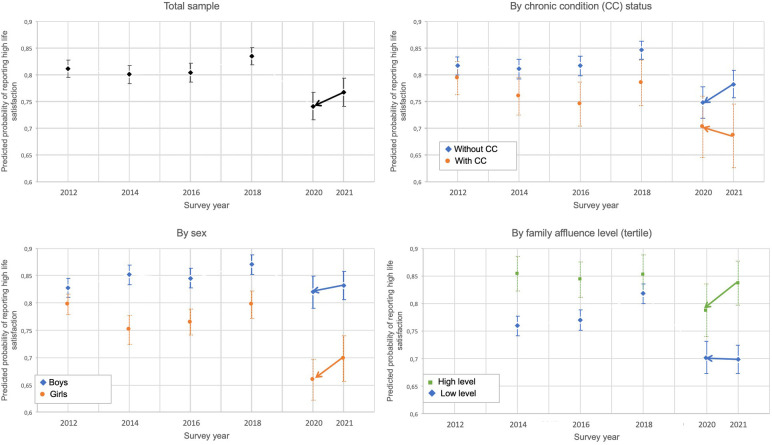
Evolution in the proportion of students with high life satisfaction between 2012 and 2021.

Table 1 presents samples characteristics and the prevalence of adolescents reporting high life satisfaction by survey year are shown in 2012, 2014, 2016, 2018 and 2021. The frequency distribution of sex was similar between survey years, while the proportion of students who reported chronic conditions, the repartition of the level of family affluence, and the proportion of students with education delay differed. From 2012 to 2018, the proportion of adolescents reporting high life satisfaction was mostly stable and then decreased from 83.8% in 2018 to 77.4% in 2021. The adjusted analyses showed that students surveyed in 2021 were less likely to report high life satisfaction than those surveyed between 2012 and 2018 (Supplementary Table S1). While sex and family affluence significantly impacted the relationship between survey years and the probability of reporting high life satisfaction (the decrease in the proportion of students reporting a high life satisfaction between 2012 and 2021 was higher in girls than boys, and higher among those from families with low affluence compared to intermediate affluence), results did not differ according to the presence of a chronic condition. The predicted probabilities of reporting high life satisfaction computed from adjusted models are graphically provided in Figure 1 in relation to the survey year. The estimate for life satisfaction in 2020 reported retrospectively by students in the 2021 survey is also reported in Figure 1.

**Table 1:** Sample characteristics and prevalence of high life satisfaction.

Survey year	2012	2014	2016	2018	2021	*p*-value^a^
	*n* = 4,887	*n* = 4,694	*n* = 3,313	*n* = 2,832	*n* = 1,960	
Age at questionnaire administration (years)^b^						
Mean (standard error)	15.1 (0.01)	15.0 (0.02)	15.1 (0.02)	15.0 (0.01)	14.7 (0.02)	<0.001
Sex, weighted %						0.306
Boys	47.8	48.7	48.4	51.1	50.7	
Girls	52.2	51.3	51.6	48.9	49.3	
Chronic condition (CC) status, weighted %						<0.001
Without CC	82.5	79.6	82.4	82.1	84.5	
With CC	17.5	20.4	17.6	17.9	15.5	
Family affluence level						0.001
Low (tertile 1)	–	43.0	47.9	46.1	43.8	
Medium (tertile 2)	–	31.6	33.6	34.1	32.2	
High (tertile 3)	–	25.4	18.5	19.8	24.1	
Educational delay, weighted %						<0.001
No	72.2	76.0	79.9	82.3	86.5	
Yes	27.8	24.0	20.1	17.7	13.5	
Life satisfaction, Cantril score						0.025
Median	7	7	7	8	7	
IQR^c^	(6–8)	(6–8)	(6–8)	(6–9)	(6–8)	
High life satisfaction^d^ (%)	81.0	80.1	80.7	83.8	77.4	

^a^ Chi-squared test with Rao & Scott's second-order correction.

^b^ In 2018, a national law changed practices in France regarding class repetition, hence educational delay.

^c^ IQR: Inter quartile range.

^d^ Cantril score ≥6.

In 2021, the sample consisted of 1,960 adolescents, of whom 49.3% were girls, 15.5% reported having a chronic condition, and 43.8% were from a family with low affluence (Table 1). Moreover, 66.8% of adolescents lived in a nuclear family and 76.7% had both parents employed (Table 2). During the 2020 lockdown, 80.9% of adolescents had their own room, 90.2% had access to an outdoor space, and 86.7% had access to a screen to contact classmates or friends online. On the other hand, almost a quarter of adolescents (22.5%) reported they could not study in good conditions during this lockdown.

**Table 2:** Socioeconomic characteristics, housing and studying conditions during the 2020 lockdown, among students enrolled in the 2021 survey.

	Total (*N* = 1,960)^a^
*Socioeconomic variables in 2021*	
Family structure, *N* (%)	
Nuclear family	1,285 (66.8)
Single parents or others	218 (10.8)
Reconstructed family	442 (22.4)
*Missing*	15
Parental employment, *N* (%)	
Both parents employed	1,320 (76.7)
One parent employed	364 (20.9)
Both parents unemployed	46 (2.5)
*Missing*	230
*Housing and studying conditions during the 2020 lockdown*	
Have had their own room: yes, *N* (%)	1,570 (80.9)
*Missing*	33
Have had access to an outdoor space (balcony, yard, patio, or garden): yes, *N* (%)	1,727 (90.2)
*Missing*	36
Have had access to a screen to contact classmates or friends online: yes, *N* (%)	1,624 (86.7)
*Missing*	71
Study conditions, *N* (%)	
Bad study conditions	430 (22.5)
Neither good nor bad study conditions	370 (19.2)
Good study conditions	1,092 (58.3)
*Missing*	68

^a^ Unweighted count (Weighted % on complete data).

### 3.2. Description of individual changes in life satisfaction between 2020 and 2021

**Figure 2:**
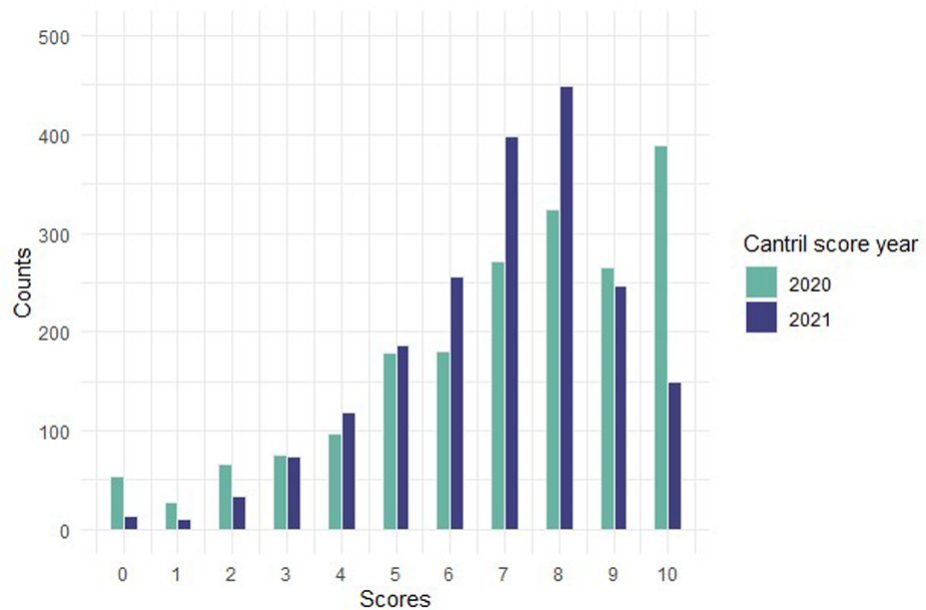
Cantril score distributions in 2020 (retrospective) and 2021.

Between 2020 and 2021, a third of the adolescents reported a change in their life satisfaction (improved: 16.3%; worsened 17.7%) (Table 3). The distribution of the Cantril score in 2021 followed an asymmetric normal distribution with a mean score of 8, whereas the distribution of the retrospective scores regarding life satisfaction during the 2020 lockdown is different, with more responses towards the extreme scores of the scale, especially 10 (Figure 2).

**Table 3:** Individual changes in life satisfaction between 2020 (retrospective) and 2021 according to housing and studying conditions during lockdown (*n* = 1,916): bivariate analysis.

	Life satisfaction scores		Individual changes between 2020 and 2021^a^			
	2020	2021	Decreased life satisfaction	No change in life satisfaction	Improved life satisfaction	*p*-value^b^
	Median (Q1–Q3)	Median (Q1–Q3)	(*n* = 337)	(*n* = 1,269)	(*n* = 310)	
Total	8 (5, 9)	7 (6, 8)	337 (17.7%)	1,269 (66.0%)	310 (16.3%)	
Sex						<0.001
Boy	8 (6, 9)	8 (6, 8)	154 (16.1%)	678 (70.9%)	123 (12.9%)	
Girl	7 (5, 9)	7 (5, 8)	183 (19.3%)	591 (60.8%)	187 (19.8%)	
Educational delay in 2021						0.200
Yes	7 (5, 9)	7 (5, 8)	46 (21.3%)	125 (59.8%)	38 (18.9%)	
No	8 (5, 9)	7 (6, 8)	291 (17.3%)	1,144 (66.7%)	272 (16.0%)	
*Missing*	* *		0	0	0	
Family structure in 2021						<0.001
Nuclear family	8 (6, 9)	7 (6, 8)	186 (14.6%)	882 (69.8%)	193 (15.6%)	
Single parents or others	7 (5, 9)	7 (5, 8)	44 (19.9%)	128 (59.6%)	45 (20.4%)	
Reconstructed family	7 (5, 9)	6 (5, 8)	104 (26.0%)	253 (57.9%)	68 (16.1%)	
*Missing*	* *		3	6	4	
Parental employment in 2021						0.500
Both parents employed	8 (6, 9)	7 (6, 8)	203 (15.6%)	895 (68.8%)	203 (15.7%)	
One parent employed	8 (5, 9)	7 (6, 8)	63 (16.9%)	231 (65.9%)	59 (17.2%)	
Both parents unemployed	7 (4, 9)	7 (4, 8)	10 (24.2%)	23 (56.8%)	7 (19.1%)	
*Missing*	* *		61	120	41	
Having a chronic condition						0.200
Yes	7 (5, 9)	7 (5, 8)	55 (21.0%)	183 (64.7%)	41 (14.4%)	
No	8 (5, 9)	7 (6, 8)	275 (17.0%)	1,074 (66.4%)	264 (16.6%)	
*Missing*	* *		7	12	5	
Have had their own room during the lockdown						0.008
Yes	8 (6, 9)	7 (6, 8)	253 (16.4%)	1,047 (67.5%)	245 (16.0%)	
No	8 (5, 9)	7 (5, 8)	82 (23.5%)	210 (59.3%)	60 (17.2%)	
*Missing*	* *		2	12	5	
Have had access to an outdoor space during the lockdown						0.700
Yes	8 (5, 9)	7 (6, 8)	302 (18.0%)	1,124 (65.5%)	279 (16.5%)	
No	8 (5, 9)	6 (5, 8)	33 (16.4%)	130 (68.6%)	27 (15.0%)	
*Missing*	* *		2	15	4	
Have had access to a screen to contact classmates or friends online during the lockdown						0.200
Yes	8 (5, 9)	7 (6, 8)	268 (16.9%)	1,076 (66.6%)	263 (16.5%)	
No	8 (5, 10)	7 (5, 8)	57 (22.1%)	162 (62.2%)	39 (15.7%)	
*Missing*	* *		12	31	8	
Study conditions during the lockdown						0.002
Bad study conditions	7 (4, 9)	6 (5, 8)	85 (20.1%)	242 (57.3%)	100 (22.7%)	
Neither good nor bad study conditions	7 (5, 9)	7 (6, 8)	59 (15.9%)	249 (67.5%)	58 (16.6%)	
Good study conditions	8 (6, 9)	8 (6, 9)	181 (17.0%)	751 (69.0%)	145 (14.0%)	
*Missing*	* *		12	27	7	

^a^ Unweighted count (Weighted %).

^b^ Chi-squared test with Rao & Scott's second-order correction.

### 3.3. Factors associated with 2020–2021 changes in life satisfaction

The comparison of changes in life satisfaction (in the 3 described groups) according to sociodemographic characteristics, housing, and studying conditions during the 2020 lockdown is presented in Table 3. The multinomial logistic regression results are presented in Table 4 for the adjusted model and in Supplementary Table S2 for the unadjusted model. Overall, girls were more likely to have experimented a change in their life satisfaction between the lockdown of 2020 and 2021 compared to boys (aOR of decreased vs. stable life satisfaction: 1.43, 95%CI, 1.14–1.79; aOR of increased vs. stable life satisfaction: 1.82, 95%CI, 1.40–2.37). Adolescents living in reconstructed families were more likely to report a decrease in life satisfaction between the 2020 lockdown period and 2021 compared to their peers living in a nuclear family (aOR 2.09, 95%CI, 1.58–2.77). There was no significant association between having a chronic condition and a change in life satisfaction. Regarding housing and studying conditions during the lockdown of spring 2020, adolescents who did not have their own room nor access to a screen to contact classmates or friends online were more likely to report a decrease in their life satisfaction between the 2020 lockdown and 2021 (aOR 1.58, 95%CI, 1.16–2.15 and aOR 1.47, 95%CI, 1.09–1.98; respectively).

**Table 4:** Association between sample characteristics and March 2020 lockdown conditions with 2021–2020 changes in life satisfaction: multinomial logistic regression analysis.

	Decreased life satisfaction 2020–2021 vs. No change		Improved life satisfaction 2020–2021 vs. No change	
	Adjusted OR^a^ (95% CI)	*p*-value	Adjusted OR^a^ (95% CI)	*p*-value
Sex				
Boy	ref		ref	
Girl	1.43 (1.14–1.79)	0.003	1.82 (1.40–2.37)	<0.001
Educational delay in 2021				
No	ref			
Yes	1.45 (0.98–2.12)	0.060	1.43 (1.00–2.04)	0.047
Family structure in 2021				
Nuclear family	ref		ref	
Single parents or others	1.58 (1.01–2.49)	0.046	1.51 (0.93–2.45)	0.092
Reconstructed family	2.09 (1.58–2.77)	<0.001	1.19 (0.87–1.64)	0.272
Parental employment in 2021				
Both parents employed	ref		ref	
One parent employed	1.11 (0.77–1.60)	0.563	1.12 (0.78–1.60)	0.531
Both parents unemployed	1.77 (0.71–4.45)	0.220	1.38 (0.42–4.56)	0.598
Having a chronic condition				
No	ref		ref	
Yes	1.26 (0.86–1.85)	0.235	0.88 (0.59–1.31)	0.517
Have had their own room during the lockdown				
Yes	ref		ref	
No	1.58 (1.16–2.15)	0.001	1.16 (0.81–1.66)	0.361
Have had access to an outdoor space during the lockdown				
Yes	ref		ref	
No	0.86 (0.55–1.32)	0.476	0.85 (0.55–1.31)	0.451
Have had access to a screen to contact classmates or friends online during the lockdown				
Yes	ref		ref	
No	1.47 (1.09–1.98)	0.011	1.10 (0.73–1.67)	0.63
Study conditions during the lockdown				
Bad study conditions	1.48 (0.99–2.21)	0.057	1.60 (0.97–2.63)	0.067
Neither good nor bad study conditions	ref		ref	
Good study conditions	1.06 (0.74–1.51)	0.750	0.84 (0.56–1.27)	0.409

^a^ Adjustment variables: sex, educational delay.

## 4. Discussion

### 4.1. Main findings

This study investigates temporal trends in life satisfaction among adolescents aged 14–15 years enrolled in middle schools in France during the COVID-19 pandemic. We found an overall slight decrease in the prevalence of adolescents reporting high life satisfaction between 2012 and 2021. The lowest proportion of adolescents reporting high life satisfaction was observed in 2021 (77.4%), with different patterns regarding sex and family affluence. Regarding individual changes in life satisfaction between the 2020 lockdown and 2021, we found that life satisfaction improved overall for 16.3% of French adolescents and decreased for 17.7%. According to adjusted models, being a girl, living in a reconstructed family (in 2021), not having their own bedroom, and not having access to a screen to contact classmates or friends online during the 2020 COVID-19 lockdown were related to a decrease in life satisfaction.

### 4.2. Interpretation

The overall decrease in life satisfaction we observed in our study between 2012 and 2021, is somewhat in line with a global decline in adolescents’ mental health, including students' life satisfaction, observed since the early 2000s' in European countries (15, 16). Studying the global burden of non-communicable diseases among adolescents in the European Union in 2019, Armocida et al. found an increase in years of life lost due to mental disorders between 1990 and 2019 (22). More recently, several systematic reviews found that the COVID-19 pandemic has impacted adolescents' mental health, with an increase in depression and anxiety symptoms and high levels of fear concerning the pandemic and its impact on their lives (3, 23). In France, a decrease in the number of self-harm acts and suicide attempts among children and adolescents was observed during the first three months of the pandemic and the first lockdown, followed by a consequent increase in the second semester of the same year (24, 25).

Among the underlying factors, sex was strongly associated with changes in life satisfaction over time. Indeed, in line with previous literature, girls are less likely to have high life satisfaction compared to boys (8, 26). Our findings confirmed this tendency for girls to report worse life satisfaction over the years, with a widening gender gap observed in 2021. Regarding the effect of the pandemic on mental health, results vary between studies. Vandentorren et al. (6) found that girls' mental health was more impacted by the pandemic in France, whereas Ravens-Sieberer et al. (2) found that in Germany, unlike in France, being a girl and older was a resource resilience factor, with fewer mental health problems. Adolescents from families from a lower socioeconomic background also appeared less likely to report high life satisfaction, as observed in other studies (27, 28). They may have had more often adverse experiences during the pandemic, such as food insecurity or a parent losing their job (29). Adverse childhood experiences were found to be associated with poorer mental health and suicidal behaviors during and after the COVID-19 pandemic (29). Although it is known that parental unemployment is related to lower life satisfaction for adolescents (30), we did not find a relationship between parental employment and changes in life satisfaction during the pandemic. In line with several previous studies (31–33), in our study, adolescents with a chronic condition were less likely to report high satisfaction than those without a chronic condition (Figure 1), whatever the survey year (Supplementary Table S2). However, our findings did not suggest a greater impact of the pandemic on changes in life satisfaction among adolescents with chronic conditions compared to adolescents without chronic conditions. Similar results were found in Australia for adolescents with neurodevelopmental disorders by Houghton et al. (34), with differences depending on the individual diagnosis. For example, adolescents with attention-deficit/hyperactivity disorder reported better well-being after school lockdown. Indeed, chronic conditions as a unique common category (i.e., without considering precise diagnosis nor the severity of the condition and its impact on schooling) could mask differences in the experience of school closures, specifically regarding working at home or being isolated from peers. For example, although not visible in our current results, we would expect to observe greater life satisfaction among adolescents with chronic conditions during lockdown because these students are more often victims of bullying (14, 35). We can also hypothesize a decrease in face-to-face bullying in 2020, but an increase in cyber-bullying.

The factors related to the housing and schooling condition during the first COVID-19 lockdown did not differ between students with stable life satisfaction and those with better life satisfaction in 2021 compared to that declared regarding the 2020 lockdown. However, the proportion of students with lower life satisfaction in 2021 compared to that reported about the 2020 lockdown was higher among students not having their own room and not having access to a screen to contact classmates or friends online. Because of the alternating opening and closing of schools (lockdown, isolation in case of COVID-19), social contacts may have been either greatly diminished regarding peers while too important within the family (making it impossible for the adolescent to have a place of their own). Here, social networks may have had a protective role on adolescents' mental health by allowing them to stay connected to their peers. Indeed, Godeau et al. observed a decrease in bullying among adolescents in France during the pandemic (36), as well as a decrease in substance use (37), which can be interpreted as a consequence of the impossibility of accessing unsupervised interactions between peers (as well as limited access to substances).

### 4.3. Strengths and limitations

The main limitation of our study is related to the retrospective data collection of the 2020 Cantril score as well as the housing and studying conditions during the March 2020 lockdown, which could be prone to recall bias. Parental employment and family structure were measured in 2021 and could have changed between 2020 and 2021. Furthermore, some other factors related to parents were not measured in this study and may have had a differential impact on the life satisfaction of adolescents since the beginning of the pandemic (e.g., domestic violence (38), life satisfaction of parents (39)). Other confounding factors could explain the differences in the fluctuation of life satisfaction between girls and boys, such as the division of domestic tasks (6).

Our study has several strengths. The EnCLASS methodology is well established, and relies on longstanding methods validated internationally (40), which was not the case with many studies published during the pandemic (5, 41). The two-stage balanced cluster sampling method made the sample representative of metropolitan French last year of middle school students. In all these surveys, the participation rate of pupils in the participating classes was around 85%. When compared to data from the Ministry of Education, each sample was representative of the targeted students (in terms of type of municipality, public-private status, sex, age). In addition, for some survey waves, calibration weights were used to further improve representativeness. Moreover, our analyses allow us to measure life satisfaction from the direct point of view of adolescents. The Cantril ladder has been used the same way in the HBSC questionnaires since 2012, and its reliability and convergent validity for adolescents have been confirmed previously (8). This allowed us to deepen and nuance the recent evolution of adolescents' perceived well-being and the impact of COVID-19 on adolescents' well-being, by putting it in a broader context.

### 4.4. Implications

Because the link between life satisfaction and mental health is no more to be questioned (42–44) and because the majority of mental disorders begin during adolescence (45), high-quality studies are needed to shed light on those and ensure mental health disorders are properly addressed by clinicians (46). Our results suggest that the identification of high-risk groups requires considering both clinical and socio-economic factors, including family structure, housing characteristics, and access to the internet. Monitoring trends in life satisfaction among adolescents can help anticipate and improve targeted prevention, screening, and medical follow-up for adolescents in need, particularly in crisis contexts such as pandemics. For example, the COVID-19 pandemic has been associated with unexpected changes in the epidemiology of adolescent suicidal behaviors in France (24, 25). However, previous studies examining such epidemiological trends were unable to evaluate the socio-economic factors that may explain the observed changes. Therefore, findings from large-scale and comprehensive studies on life satisfaction, such as the EnCLASS study, straighten the need to better support school health services, including after long periods of school absence (47). Furthermore, life satisfaction assessments offer an opportunity for pediatric and child health professionals to provide care services with a global approach of adolescents' health.

### 4.5. Conclusions

With a nationally representative sample and validated methods (compared to ad-hoc surveys), our study shows that the way French 14–15 years old adolescents experienced the first 2020 lockdown was not unequivocal, even if the majority reported no significant difference in their life satisfaction between 2020 and 2021. These results illustrate the complexity of measuring the impact of the pandemic on the health and well-being of adolescents, and the need to better understand associated factors. While the focus of this study was the changes between 2020 and 2021, more international research is needed to assess the long-term effect of the COVID-19 pandemic on adolescents in order to support them efficiently, even more if they belong to vulnerable groups. Such findings can help clinicians to identify adolescents at high risk of mental health problems in their practice. Our results should also help develop health promotion programs enhancing child well-being and life satisfaction.

## Data Availability

The datasets presented in this article are not readily available because the data are made available upon request 3 years after the data collection. Requests to access the datasets should be directed to EG - emmanuelle.godeau@enclass.fr.
