# Supplementary figures and images for: Life satisfaction in the context of the COVID-19 pandemic among middle school adolescents in France: findings from a repeated cross-sectional survey (EnCLASS, 2012–2021)
Source: Front Pediatr. 2023 Aug 8;11:1204171. doi: 10.3389/fped.2023.1204171 (PMC10443644; doi:10.3389/fped.2023.1204171)

## Supplementary Figure 1: Chronology of school closures in France in 2020 et 2021

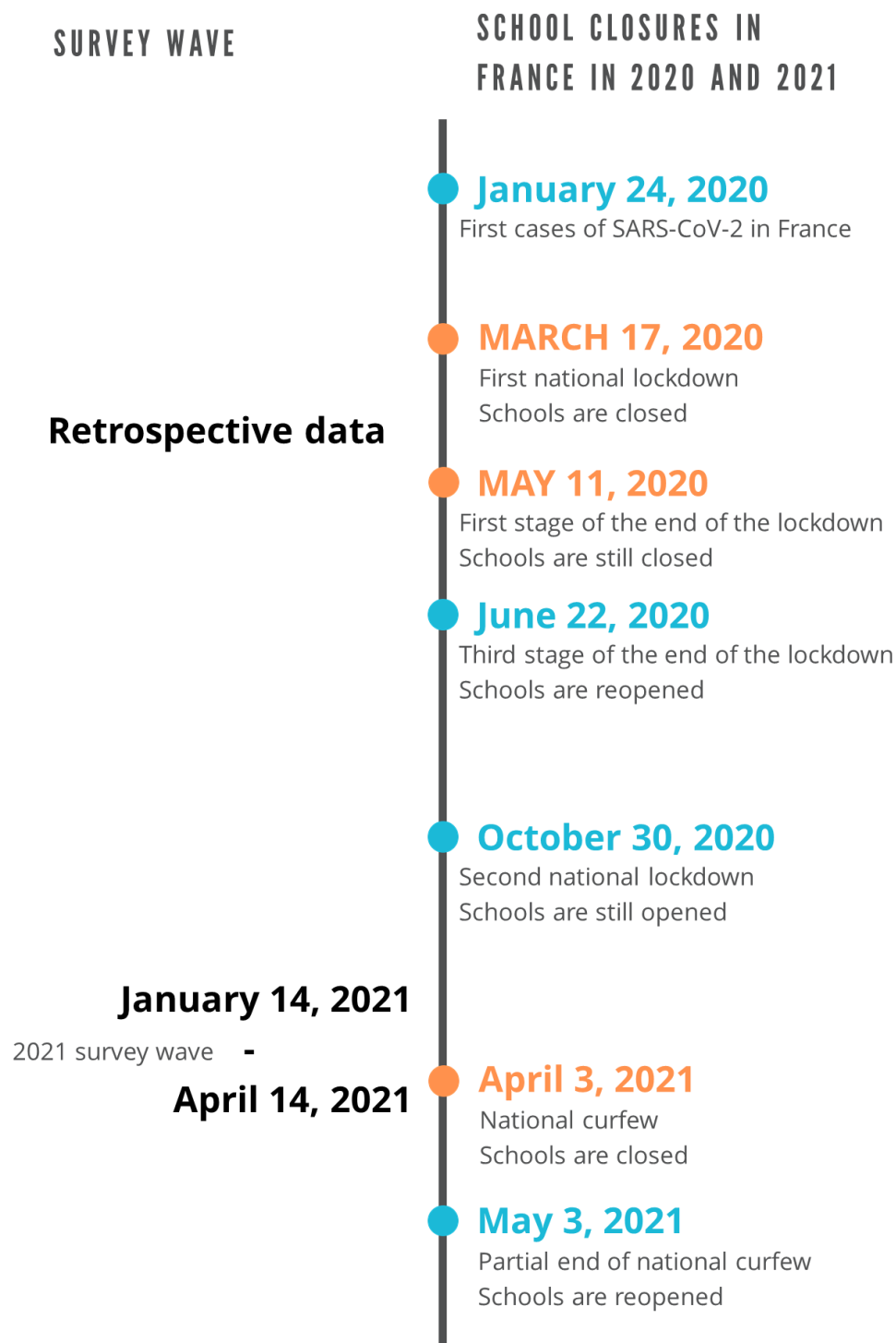

Supplement: Supplementary file 1 [file Image1.pdf]
