# Supplementary material for: Life satisfaction in the context of the COVID-19 pandemic among middle school adolescents in France: findings from a repeated cross-sectional survey (EnCLASS, 2012–2021)
Source: Front Pediatr. 2023 Aug 8;11:1204171. doi: 10.3389/fped.2023.1204171 (PMC10443644; doi:10.3389/fped.2023.1204171)
Supplement: Supplementary file 2 [file Table1.docx]

Supplementary Table 1: Adjusted analysis between 2012 and 2021, modeling the prevalence rate of high life satisfaction: prevalence ratio (PR) between survey years

|  | **2012** | **2014** | **2016** | **2018** | **2021** |
| --- | --- | --- | --- | --- | --- |
|  | Adjusted PR | Adjusted PR | Adjusted PR | Adjusted PR | Adjusted PR |
|  | (95%CI) | (95%CI) | (95%CI) | (95%CI) | (95%CI) |
| **Model with no interaction term†** | | |  |  |  |
|  | 1.06 | 1.04 | 1.05 | 1.09 | ref |
|  | (1.03,1.09) | (1.01,1.07) | (1.02,1.07) | (1.05,1.12) |  |
| **Model with interaction between chronic condition (CC) status and survey year †‡** | | | | | |
| Without CC | 1.04 | 1.04 | 1.04 | 1.08 | ref |
|  | (1.01,1.08) | (1.00,1.07) | (1.01, 1.08) | (1.04,1.12) |  |
| With CC | 1.15 | 1.10 | 1.08 | 1.14 | ref |
|  | (1.05,1.26) | (1.00,1.22) | (0.98,1.19) | (1.03,1.26) |  |
| Interaction term ( p-value) | 0.041 | 0.221 | 0.462 | 0.304 |  |
| **Model with interaction between sex and survey year †‡** | | | |  |  |
| Boys | 0.99 | 1.02 | 1.02 | 1.04 | ref |
|  | (0.96,1.03) | (0.99,1.06) | (0.98,1.05) | (1.01,1.08) |  |
| Girls | 1.14 | 1.07 | 1.10 | 1.14 | ref |
|  | (1.08,1.19) | (1.02,1.13) | (1.04,1.15) | (1.08,1.19) |  |
| Interaction term ( p-value) | 0.000 | 0.186 | 0.023 | 0.011 |  |
| **Model with interaction between family affluence level and survey year †‡** | | | | |  |
| Low level (tertile 1) | n/a | 1.08 | 1.10 | 1.17 | ref |
|  |  | (1.02,1.15) | (1.04,1.16) | (1.11,1.24) |  |
| High level (tertile 3) | n/a | 1.02 | 1.01 | 1.02 | ref |
|  |  | (0.97,1.08) | (0.95,1.07) | (0.96,1.08) |  |
| Interaction term ( p-value) | n/a | 0.140 | 0.028 | 0.001 |  |

**†** Poisson regression model with robust variance, with high life satisfaction as the outcome variable. All the models controlled for school delay (yes/no).

**‡** As the model includes an interaction term, the estimates are presented by subgroups

Abbreviation: n/a=not available
