# Supplementary material for: Life satisfaction in the context of the COVID-19 pandemic among middle school adolescents in France: findings from a repeated cross-sectional survey (EnCLASS, 2012–2021)
Source: Front Pediatr. 2023 Aug 8;11:1204171. doi: 10.3389/fped.2023.1204171 (PMC10443644; doi:10.3389/fped.2023.1204171)
Supplement: Supplementary file 3 [file Table2.docx]

Supplementary Table 2: Results from the unadjusted multinomial regression estimating the Odd Ratios for the association between sample characteristics and March 2020 lockdown conditions with 2021-2020 changes of life satisfaction

|  |  | **Unadjusted models** | | | |
| --- | --- | --- | --- | --- | --- |
|  |  | **Decreased life satisfaction** | | **Improved life satisfaction** | |
|  |  | OR (95% CI) | p-value | OR (95% CI) | p-value |
| ***Sociodemographic characteristics*** | |  |  |  |  |
| **Sex** | |  |  |  |  |
|  | Boy | ref |  | ref |  |
|  | Girl | 1.35 (1.07-1.71) | 0.012 | 1.75 (1.34-2.29) | < 0.001 |
| **School delay in 2021** | |  |  |  |  |
| No | | ref |  | ref |  |
| Yes | | 1.37 (0.94-2.01) | 0.099 | 1.31 (0.92-1.86) | 0.130 |
| **Family structure in 2021** | |  |  |  |  |
|  | Nuclear family | ref |  | ref |  |
|  | Single parents or others | 1.65 (1.04-2.61) | 0.033 | 1.51 (0.95-2.40) | 0.081 |
|  | Reconstructed family | 2.19 (1.69-2.84) | < 0.001 | 1.26 (0.92-1.72) | 0.142 |
| **Parental employment in 2021** | |  |  |  |  |
|  | Both parents employed | ref |  | ref |  |
|  | One parent employed | 1.15 (0.81-1.64) | 0.430 | 1.14 (0.81-1.61) | 0.454 |
|  | Both parents unemployed | 1.85 (0.73-4.67) | 0.190 | 1.47 (0.49-4.43) | 0.489 |
| **Having a chronic condition** | |  |  |  |  |
| No | |  |  |  |  |
| Yes | | 1.27 (0.88-1.83) | 0.200 | 0.88 (0.60-1.30) | 0.523 |
| ***Housing and studying conditions during lockdown*** | |  |  |  |  |
| **Have had their own room** | |  |  |  |  |
| Yes | | ref |  | ref |  |
| No | | 1.65 (1.23-2.21) | 0.001 | 1.23 (0.89-1.72) | 0.208 |
| **Have had access to an outdoor space** | |  |  |  |  |
| Yes | | ref |  | ref |  |
| No | | 0.85 (0.53-1.37) | 0.503 | 0.85 (0.53-1.37) | 0.508 |
| **Have had access to a screen to contact classmates or friends online** | |  |  |  |  |
| Yes | | ref |  | ref |  |
| No | | 1.41 (1.01-1.96) | 0.041 | 1.05 (0.71-1.56) | 0.805 |
| **Study conditions** | |  |  |  |  |
|  | Bad study conditions | 1.48 (0.99-2.20) | 0.054 | 1.61 (0.97-2.66) | 0.063 |
|  | Neither good nor bad study conditions | ref |  | ref |  |
|  | Good study conditions | 1.06 (0.74-1.50) | 0.753 | 0.84 (0.56-1.25) | 0.381 |

Ref: reference category
